# 3D Tortuosity computation as a shape descriptor and its application to brain structure analysis

**DOI:** 10.1186/s12880-024-01312-6

**Published:** 2024-06-04

**Authors:** Maria-Julieta Mateos, Ernesto Bribiesca, Adolfo Guzmán-Arenas, Wendy Aguilar, Jorge A. Marquez-Flores

**Affiliations:** 1 Graduate Program in Computer Science and Engineering, Universidad Nacional Autónoma de México, Mexico City, México; 2 Institute of Research in Applied Mathematics and Systems (IIMAS), Universidad Nacional Autónoma de México, Circuito Escolar 3000, Ciudad Universitaria, 04510 Coyoacán, Mexico City, México; 3 Centro de Investigación en Computación, Instituto Politécnico Nacional, Mexico City, México; 4 Instituto de Ciencias Aplicadas y Tecnología, Universidad Nacional Autónoma de México, Universidad Nacional Autónoma de México, Circuito Exterior S/N, Ciudad Universitaria, 04510 Mexico City, México

**Keywords:** 3D tortuosity, Brain morphology, Alzheimer’s disease, Discrete tortuosity

## Abstract

In this study, we propose a novel method for quantifying tortuosity in 3D voxelized objects. As a shape characteristic, tortuosity has been widely recognized as a valuable feature in image analysis, particularly in the field of medical imaging. Our proposed method extends the two-dimensional approach of the Slope Chain Code (SCC) which creates a one-dimensional representation of curves. The utility of 3D tortuosity ($\tau _{3D}$) as a shape descriptor was investigated by characterizing brain structures. The results of the $\tau _{3D}$ computation on the central sulcus and the main lobes revealed significant differences between Alzheimer’s disease (AD) patients and control subjects, suggesting its potential as a biomarker for AD. We found a $p<0.05$ for the left central sulcus and the four brain lobes.

## Introduction

The representation of three-dimensional data, objects, and images in digital form is a fundamental element in a wide range of fields, such as manufacturing, architecture, video games, medicine, geography, and biology. One of the major benefits of using these digital representations is the ability to measure and compare different characteristics and properties of the objects, as noted for example by [1] and [2]. As a result, researchers have shown a great deal of interest in modeling and quantifying the shape of three-dimensional images, as evidenced by studies such as those by [3] and [4]. This interest is particularly relevant in medical imaging, where automatic segmentation of different body structures is an area of focus, and the morphological characteristics of the images are valuable. For instance, [5] used fractal dimension analysis to examine the shape of the cerebellum, which led to a deeper comprehension of Chiari malformation type I.

Tortuosity, a morphological property that reflects the complexity of objects and that is defined in the Merriam-Webster dictionary as “tortuos” as “full of twists, turns; crooked” [6], is one such property that has been measured in diverse fields. Its applications include detecting certain conditions in retinal images [7], characterizing and modeling rivers [8], quantifying morphological changes of blood vessels [9], and in airborne ultrasound to measure tortuosity in aluminum foams [10].

**Fig. 1:**
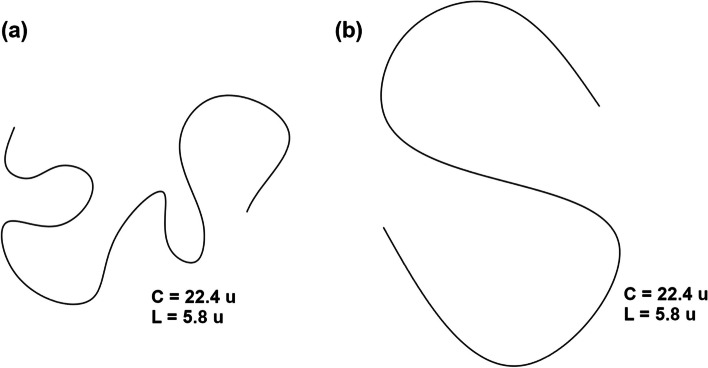
Two curves with identical arc length and underlying chord, but with different morphological characteristics. Despite having the same tortuosity value, curve (**a**) has more twists and turns than curve (**b**), demonstrating the limitations of using the arc length to chord length ratio approach to capture the morphological differences of shapes

Measuring the tortuosity of vessels is a common practice, and the classical method widely used for this purpose is to calculate the ratio between the arc length of the curve (*C*) and the length of the underlying chord (*L*), denoted as $\tau = C/L$, according to Lotmar [11]. One drawback of the arc length to chord length ratio approach is that it fails to fully capture the shape of curves. As an example, consider Fig. 1, which shows two curves with different shapes and turns, but the same length of curve and underlying chord, resulting in the same tortuosity value. However, curve *(a)* has significantly more twists and turns than curve *(b)*, making it more tortuous. As a result, this definition does not account for these morphological differences, highlighting the limitations of this approach.

Several methods have been proposed to measure tortuosity in 2D structures, including approaches based on angle changes at discrete steps [12], curvature by segments [13], integration of all direction changes [14], evaluation of the number of inflection points to distinguish smoothly curved vessels from those with abrupt changes in direction [9], a novel automatic grading method for retinal vessel tortuosity [15], and more recently, Bribiesca [16] proposed a tortuosity measure for 2D curves represented by means of the Slope Chain Code (SCC).

The use of SCC as a representation for 2D curves provides important advantages for computing tortuosity, as it is independent of translation, rotation, and scaling. Moreover, this approach has shown promising results in high-definition contour shapes, as demonstrated in [17], and the application of grammatical techniques simplifies the tortuosity computation process. For a review of techniques used to measure the tortuosity of retinal blood vessels, please refer to [18].

Extending the measurement of tortuosity from the 2D to 3D domain is critical as it has significant applications in studying the morphology of surfaces and 3D objects. Additionally, incorporating tortuosity as an extra property can facilitate the classification of these types of structures. However, only a few authors have attempted to measure 3D tortuosity, making it an area with considerable scope for research.

For example, Nemati et al. [19] used a stochastic approach to predict tortuosity in porous media through pore network modeling. Lala [20] developed a method for estimating tortuosity in reservoir rocks, while Ylyasova [21] presented several techniques for evaluating 3D features of blood vessels. Malek et al. [22] investigated the impact of retinal vascular tortuosity on retinal circulation, and Ramachandran [23] introduced U-COSFIRE filters for quantifying vessel tortuosity. Zhang and Nagy [24] proposed three measures of tortuosity to analyze cracks in concrete, but their results only show that the measurements can differentiate surfaces without explaining which one better represents the morphological changes. Zhang and Nagy [24] proposed three measures of tortuosity to analyze cracks in concrete: 1) the average angle between surface normals, 2) the average principal curvatures, and 3) the standard deviations of principal curvatures. While their work demonstrated that these tortuosity measures can effectively differentiate surfaces, they did not provide insight into which measure is best suited to capture morphological changes. Finally, W. Xiao et al. [25] explored the combined impact of tortuosity and surface roughness on the estimation of flow rate through a single rough joint. Their study analyzed how fluid flow through rock joints is significantly influenced by both factors. However, in order to carry out this analysis, it is necessary to obtain the 3D object meshes through data interpolation.

More recently, a measure of tortuosity for enclosing surfaces was proposed [26]. This measure is based on the relation between three surfaces: the enclosing surface area (*A*), defined as the sum of areas of the external plane faces of the voxels forming the visible faces of the solid; the contact surface area ($A_{c}$), which is the sum of the areas of the contact surfaces which are common to two voxels; and the total surface area ($A_{t}$), defined as the sum of all the surface areas of the faces of all voxels of the solid. This measure of tortuosity is related to compactness, another discrete measure to describe surfaces, and is not meant to characterize the shape of the voxelized objects.

In this paper, we introduce a novel approach to measure the tortuosity of 3D voxelized objects, which extends the two-dimensional SCC proposal by Bribiesca [16] into three dimensions. The proposed method can effectively describe and characterize a wide variety of voxelized objects, such as tumors, organs, brain structures, archaeological artifacts, and bones, while also offering the advantage of utilizing data directly extracted from the voxelized objects, without requiring interpolation. Our goal is to provide a straightforward approach for capturing and measuring morphological features of different 3D objects. Some of the main advantages of this proposal are its simplicity and the amount of time it takes to compute tortuosity compared to surface-based descriptors. A first attempt to compute the three-dimensional tortuosity by our group is presented [27]. Here we present a full description of the method, its validation, and more examples of its potential use as a biomarker for neurodegenerative diseases.

### Concepts and definitions

In this section, we present key concepts and definitions that are relevant for describing our proposed method for measuring the tortuosity of voxelized objects.

### Curvature

A curve is a fundamental mathematical concept that is defined as a continuous function that maps a one-dimensional space to an n-dimensional space. The representation of the boundaries of real-world planar objects using a category of planar curves and arcs was introduced by Latecki and Rosenfeld [28]. Curvature is another essential concept that allows the characterization of curves. It is defined by James and James [29] as “the absolute value of the rate of change of the angle of inclination of the tangent line with respect to distance along the curve”. In other words, curvature provides a measure of the amount of bending in a curve. For example, in the case of a circle, the curvature is the reciprocal of the radius, which is a well-known property of this geometric figure.

**Fig. 2:**
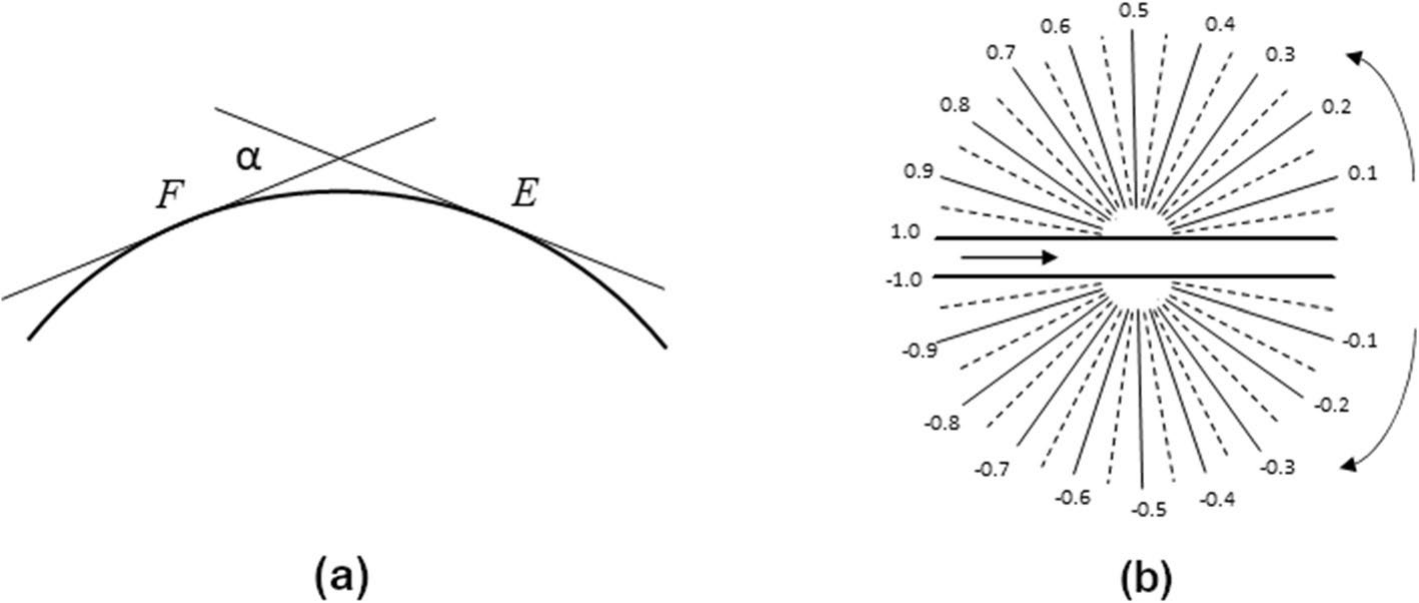
Curvature: **a** continuous curvature; **b** the range of slope changes [0, 1) and [$0,-1$)

The ratio between the angle of contingency $\alpha$ and the length of an arc *EF* (see Fig. 2a) represents the average curvature $K_{av}$ of the arc. This average curvature is equivalent to the geodesic distance between the points of the arc *EF*, as shown in Eq. 1.

1 $$\begin{aligned} K_{av} = {\alpha \over {EF}} \end{aligned}$$

The curvature $K_E$ of a line at a given point *E* is the limit of the average curvature of arc *EF*, when the length of this arc approaches zero (that is, when point *F* approaches point *E*) and is defined as follows:

2 $$\begin{aligned} K_{E} = \underset{F\rightarrow E}{\lim} K_{av}= \underset{EF\rightarrow 0}{\lim} {\alpha \over {EF}} \end{aligned}$$

### Discrete curvature

When dealing with curves in a discrete space, we can make certain assumptions about their geometry. For instance, we can assume that the arc *EF* is constant and straight, as in the notation of the SCC [16]. This assumption simplifies the calculation of the average curvature $K_{av}$. To be more specific, if we set *EF* equal to 1 in Eq. 1, then:

3 $$\begin{aligned} K_{E} = \alpha . \end{aligned}$$

Therefore, the curvature of a discrete curve located at the center point *G* is defined as the angle of contingency $\alpha$, or the slope change between contiguous straight-line segments at that point. This is known as the *discrete curvature* of the curve. To keep the values within a range of ($-1,1$), the slope change is normalized. Extreme values of 1 or $-1$ are not taken into consideration for practical purposes. The interval [0, 1) and [$0,-1$) that define the range of slope changes is depicted in Fig. 2b.

### Slope chain code

The chain code is a contour-based representation that captures the boundaries of a region and results in lossless data reduction of the image. It is determined by selecting a starting pixel and encoding the sequence of moves along the boundary to reach the next pixel [30].

According to [16], a chain denoted as *A* can be described as a sequence of *n* ordered elements, which is expressed using Eq. 4

4 $$\begin{aligned} A=a_{1} a_{2} \ldots a_{n} \end{aligned}$$

The Slope Chain Code (SCC) is a type of chain where the element $a_n$ represents the slope change between contiguous straight-line segments of the curve in that element position. It is important to note that the range of slope changes in SCC is between -1 and 1.

### 2D Tortuosity

The degree of turns or detours in objects can be measured using tortuosity $\tau$, which is a metric for calculating deformations in different objects. In this study, the SCC (Eq. 5) is used as the basis for this shape measurement, where $\tau$ is determined by summing up all the absolute values of the chain elements $a_{n}$ and *N* represents the total number of elements [16].

5 $$\begin{aligned} \tau = \sum \limits _{n=1}^{N} |a_{n} |\end{aligned}$$

## Methods

In this section, we describe the proposed methods for measuring tortuosity using a voxelized representation of a three-dimensional object as input.

### Measure of tortuosity for 3D objects

The proposed method consists of four sequential steps for measuring tortuosity:

1. Obtain the voxelized representation of the object of interest.
2. Track contours for every slice *i*, *j*, *k*, for each corresponding direction *X*, *Y*, *Z*
3. Filter the stair-stepping artifact.
  Downsample the contours.
  Apply the Digital Straight Segment (DSS) algorithm [31].
4. Tortuosity measure for 3D objects ($\tau _{3D}$).

#### Voxelized representation

A discrete representation of three-dimensional objects can be obtained either by voxelization or 3D reconstruction from multiple 2D images, where the voxel serves as the basic volume unit. Voxelization is the process of approximating *continuous* geometric data structures by a set of voxels. This results in the data of the object being stored in a regular, discrete 3D grid [32]. On the other hand, 3D reconstruction from multiple 2D images is a mathematical process that generates volumetric models or “3D images”. The specific 3D reconstruction techniques used depend on the acquisition methods employed. The use of this type of representation has become essential for a wide range of applications in various fields, including medicine (such as Magnetic Resonance Imaging, Computerized Tomography, and Positron Emission Tomography), video games, robotics, augmented reality, computer vision, and many others [33].

#### Contour tracking

After obtaining the voxelized representation of the object of interest, the next step is to approximate its boundary by using straight-line segments for each slice $s_{i}$ in the *X*, *Y*, and *Z* directions. This is achieved through a process called contour tracking, which traces the complete border of the object to obtain a sequence of boundary points without vertex repetition. Two distinct approaches for contour tracking are described in [34] and [35].

#### Filtering the stair-stepping artifact

A significant drawback of utilizing voxelized representations is the existence of artifacts caused by stair-stepping. These artifacts affect the measurement of tridimensional tortuosity $\tau _{3D}$ as they make it challenging to obtain accurate slope changes that correspond to the object’s morphology.

**Fig. 3:**
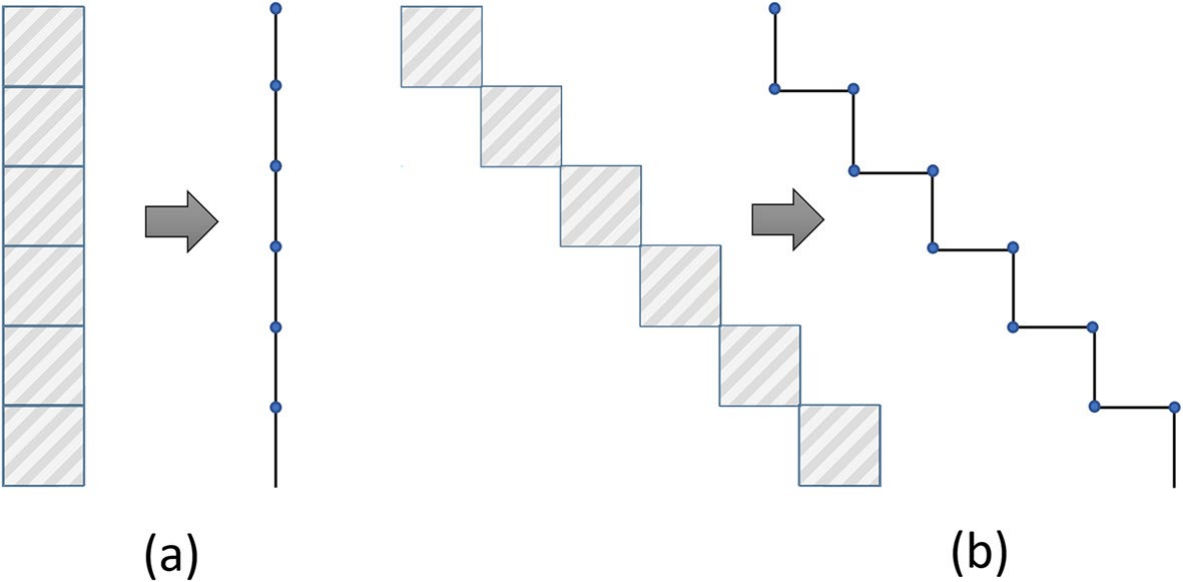
Example of the stair-stepping artifact. **a** Voxelized representation of a straight line in a 90 degree angle and the corresponding contour. **b** Voxelized representation of a straight line in a 45 degrees angle and the corresponding contour

Figure 3a illustrates the stair-stepping problem. It shows a straight line represented in a voxelized format, viewed from two distinct angles along with its corresponding contour. When the angle of the line deviates from 0 or 90 degrees, as demonstrated in Fig. 3b, a stair-stepping error becomes apparent.

In order to minimize the contribution of the stair-stepping artifact and obtain an accurate depiction of the voxelized object’s morphology, we designed a two-step filter. The first step involves downsampling the contour vertices, followed by the application of a DSS algorithm.

- ***Downsampling contours.*** To mitigate the error caused by stair-stepping artifacts, this step involves decreasing the sampling frequency of the tracked contours by a factor of ten. The choice of downsampling factor (DSF) is influenced by the size of the analyzed objects and is used to balance the reduction of the artifact with the preservation of relevant details in the morphology of the voxelized object.
- ***Digital straight segment algorithm.*** Once the contours have been downsampled, a DSS-algorithm is applied to select the vertices that define different straight segments. DSS-algorithms use a polygonal approximation to curves [36]. For this work, the Kovalevsky method [37] has been selected, which is based on calculating the narrowest strip defined by the nearest support below and above.

#### Tortuosity measure for 3D objects

The proposed 3D tortuosity measure extends the 2D measurement based on SCC by summing up all the slope changes in each contour across the three directions.

The following equations, expressed below as Eq. 6, represent the 3D adaptation of the chain element (as seen in Eq. 4). In these equations, *N* denotes the total number of chain elements, and $S_{X}$, $S_{Y}$, and $S_{Z}$ denote the number of slices in each direction.

The relationship between 2D tortuosity and 3D tortuosity computed per slice (*i, j, k*) is presented in Eq. 7. To compute $\tau _{3D}$, the proposed equation is presented in Eq. 8, where $N_{i}, M_{j}, L_{k}$ refer to the number of chain elements and slope changes for each slice and direction.

The proposed equation to compute $\tau _{3D}$ is presented in Eq. 8, where $N_{i}, M_{j}, L_{k}$, are the number of chain elements, and slope change for each slice, and direction.

6 $$\begin{aligned} X_{i}=x_{1i} x_{2i} \ldots x_{Ni},\ \text{contour(s)in slice}\ i,\ \text{direction}\ X, i &= {} 1,\ldots , S_{X}\nonumber \\ Y_{j}=y_{1j} y_{2j} \ldots y_{Mj},\ \text{contour(s)in slice}\ j,\ \text{direction}\ Y, j &= 1,\ldots , S_{Y}\nonumber \\ Z_{k}=z_{1k} z_{2k} \ldots z_{Lk},\ \text{contour(s)in slice}\ k,\ \text{direction}\ Z, k &= 1,\ldots , S_{Z} \end{aligned}$$

7 $$\begin{aligned} \sum \limits _{n=1}^{N_{i}}\mid {a_{n}}\mid \rightarrow \sum \limits _{n=1}^{N_{i}}\mid {x_{ni}}\mid ,\sum \limits _{m=1}^{M_{j}}\mid {y_{mj}}\mid ,\sum \limits _{l=1}^{L_{k}}\mid {z_{lk}}\mid \end{aligned}$$

8 $$\begin{aligned} {\tau _{3D}}=\frac{\sum \limits _{i=1}^{{S_{X}}}\sum \limits _{n=1}^{N_{i}}\mid {x_{ni}}\mid }{{S_{X}}}+\frac{\sum \limits _{j=1}^{{S_{Y}}}\sum \limits _{m=1}^{M_{j}}\mid {y_{mj}}\mid }{{S_{Y}}}+\frac{\sum \limits _{k=1}^{{S_{Z}}}\sum \limits _{l=1}^{L_{k}}\mid {z_{lk}}\mid }{{S_{Z}}} \end{aligned}$$

### Validation and error estimation

Based on Bribiesca’s proposal, the tortuosity $\tau$ value for simple convex closed curves always equals 2 [16], extending it to surfaces the value of $\tau _{3D}$ for convex closed surfaces is always 6. This theoretical framework also implies that the tortuosity measurement is invariant under scaling. All of which allows the validation of the proposed method and its error estimation. For this purpose, a group of voxelized spheres (convex, closed surfaces) was generated at different angles and with different radii.

**Fig. 4:**
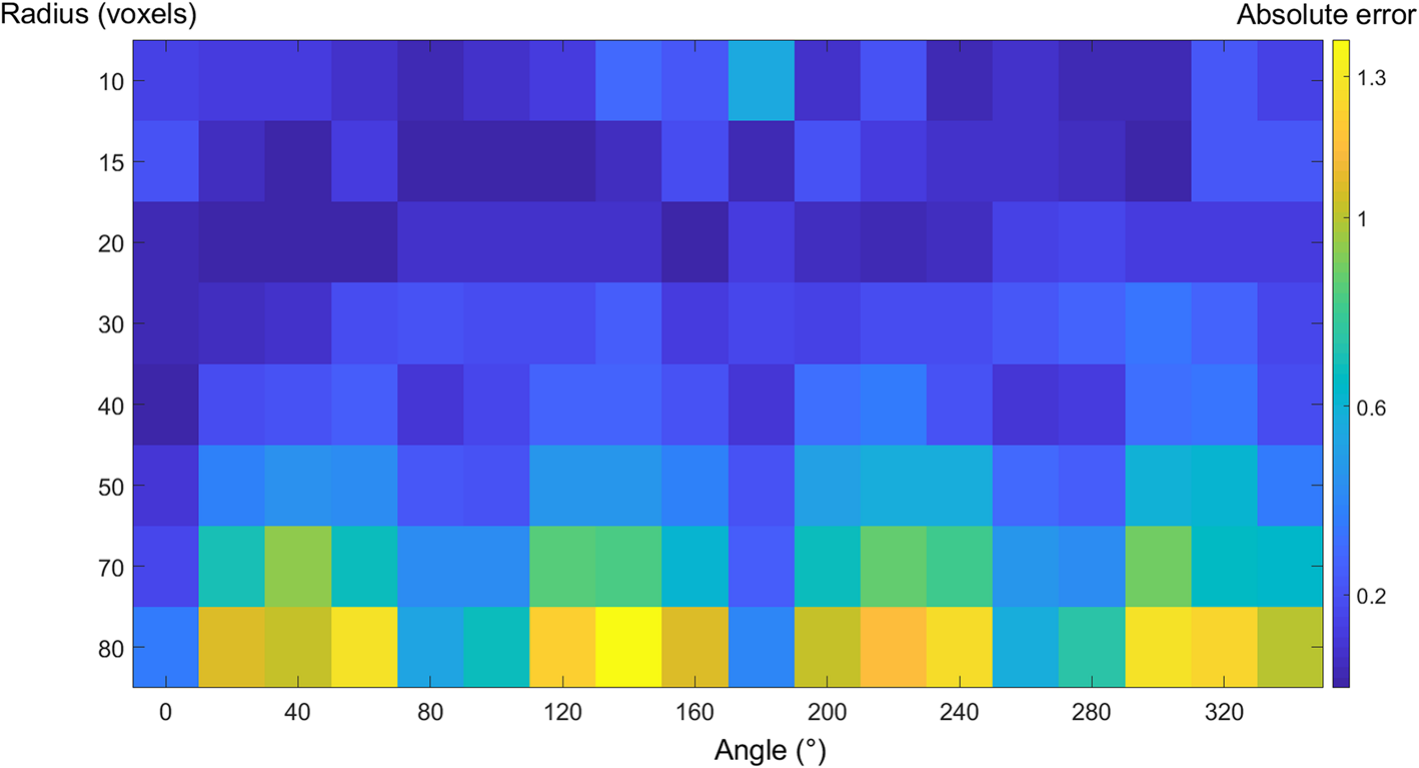
Tortuosity error for different radii and angles. The Y-axis shows different radius sizes (voxels), X-axis, and generating angle; the color-scale shows the absolute error of measured tortuosity. The darker the color the less absolute error

The $\tau _{3D}$ value was measured for each sphere to determine the accuracy and the absolute error ($\Delta x$). In Eq. 9, $x_{i}$ represents each obtained value of $\tau _{3D}$, while *x* represents the theoretical value (for a sphere, $\tau _{3D} = 6$). Figure 4 presents the $\Delta x$ of the computed values of tortuosity as the radius and angle change. The X-axis represents the different angles at which the sphere is generated, ranging from 0 to 360 degrees, while the Y-axis shows various spheres radii, ranging from 10 to 80 voxels. The color bar indicates the absolute error of $\tau _{3D}$. It is noteworthy that the tortuosity can be computed with an error of ± 1 for objects with $r \in [10, 70]$ voxels. As the radius of the voxelized sphere increases, the relationship between the voxel size and the sphere’s curvature undergoes a significant change. Due to the constant downsampling factor used in these experiments, the downsampling process becomes insufficient in filtering out stair-stepping artifacts for larger spheres radii. Thus, these artifacts can introduce inaccuracies in the voxelized representation, contributing to the observed increase in error ($\Delta x$). These results suggest that the proposed method for computing tortuosity is, to some extent, invariant under scaling for a downsampling factor of 10 (as shown in Fig. 4). It is important to note that the computation of $\tau _{3D}$ is extremely sensitive to the definition of chain elements; thus, the filtering process is essential to achieve an accurate estimation.

9 $$\begin{aligned} \Delta x= |x_{i}-x |\end{aligned}$$

## Results: brain morphometry applications of $\boldsymbol{\tau}_{\boldsymbol{3D}}$  

To appreciate the potential and possible applications of the 3D tortuosity to quantify the shape of different brain structures, we measure the $\tau _{3D}$ of the brain in two different scenarios.

### Pial surface

The first application consisted in using the proposed method to measure the $\tau _{3D}$ of the brain’s pial surface employing different levels of smoothing filters. Here, a morphological closing operation was performed on a binary image *I* using a structuring element *B* as the smoothing filter, as defined in Eq. 10. The dilation operator $\oplus$ and the erosion operator $\ominus$ are used to fill holes, reduce concavities, and smooth rough features.

Thus, it is expected that the tortuosity of the voxelized pial surface of the brain will decrease as the structural element *B* increases during the smoothing process. To evaluate whether the proposed method behaves as expected, a discrete sphere with various radii ($r\in {2,4,6,8}$) was used as the structuring element *B* in the morphological closing operation applied to the original structure.

**Fig. 5:**
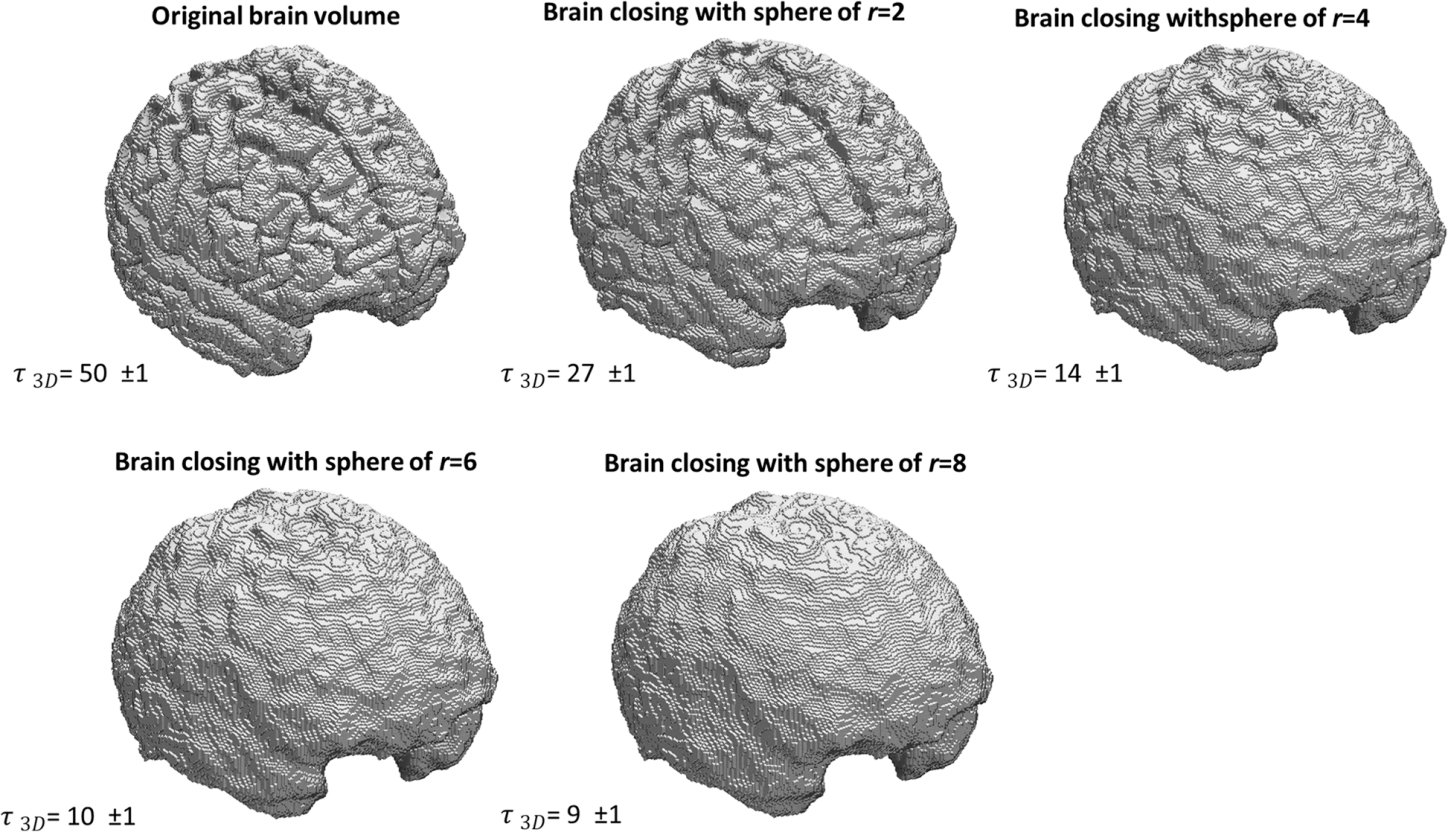
Brain volumes after applying the mathematical morphology operator of “closing” (Eq. 5) with five structuring elements: a sphere with increasing radii, and the corresponding computed values of 3D tortuosity

In Fig. 5, we can observe a loss of surface details (in the gyri and the sulci) as the radius of the structuring element *B* increases. We can also appreciate, as expected, that the tortuosity $\tau _{3D}$ decreases with the increasing size of *B*. The last two volumes exhibit no statistically significant difference in tortuosity values. The sensitivity of $\tau _{3D}$ becomes apparent in these results.

10 $$\begin{aligned} (I\oplus B)\ominus B \end{aligned}$$

### Alzheimer’s disease brain morphological changes: brain lobes

Alzheimer’s disease is a neurodegenerative disorder that is known to cause significant grey matter loss in various brain regions [38, 39]. This loss is expected to result in morphological changes in the pial surface of the brain. To assess these changes, our group previously proposed a method to estimate the sulcal width [40], which revealed that the mean sulcal width is typically greater for Alzheimer’s patients across most brain sulci.

**Fig. 6:**
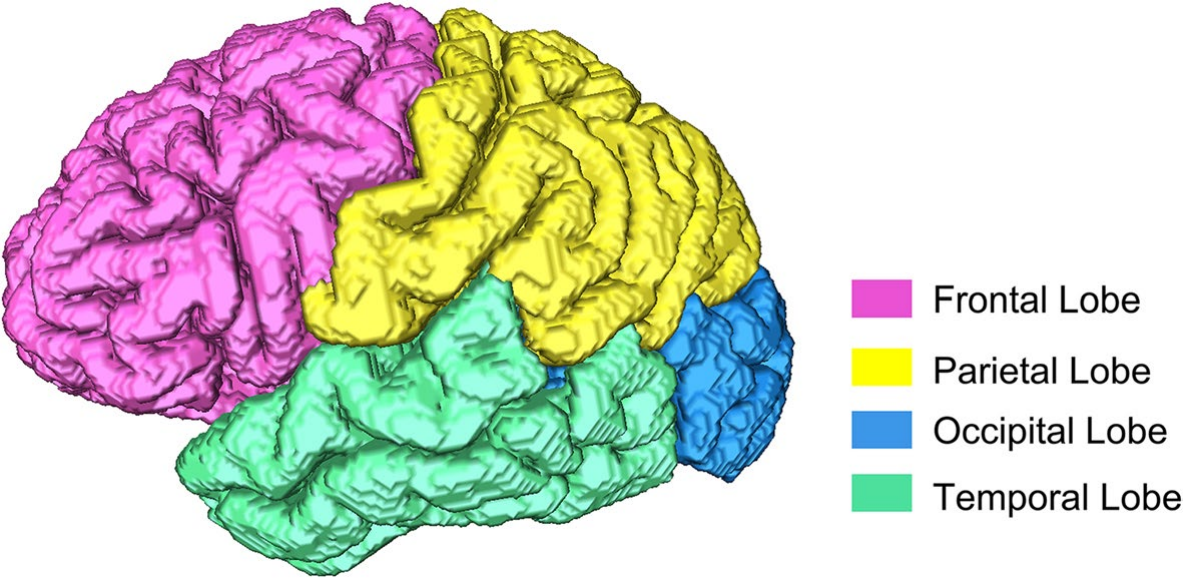
Brain surface divided into the temporal, parietal, occipital, and temporal lobes

In this section, the goal is to investigate whether the proposed method can detect and quantify the aforementioned morphological changes. To this end, we utilized images from the Minimal Interval Resonance Imaging in Alzheimer’s Disease (MIRIAD) database, comprising high-resolution MRI scans of 69 subjects. Given the sensitivity of 3D to both image quality and segmentation accuracy, a visual inspection was conducted. This involved a thorough inspection of the initial T1-weighted (T1w) images and the resultant brain extractions. Subsequently, nine subjects were identified as not meeting the criteria. As a result, our study focused on a subset of 60 subjects, including 37 patients diagnosed with Alzheimer’s Disease (AD) and 23 control subjects. In our previous work [27] the $\tau _{3D}$ values were measured for the central sulcus and the results showed that the values were significantly greater, with a z-value of 2.32, for the AD patients ($p< 0.05$) for the left hemisphere. In contrast, the tortuosity values obtained for the central sulci on the right hemisphere cannot be differentiated between AD patients and control subjects. In this work, we segmented the frontal, temporal, parietal, and occipital lobes of the brain, and $\tau _{3D}$ was calculated for each of them. An example of the segmentation result in one of the subjects is presented in Fig. 6.

Table 1 presents the $\tau _{3D}$ values calculated for all lobes and patients. Afterward, we used a Wilcoxon test to compare the differences between Alzheimer’s patients and control subjects. The results showed that there were statistically significant differences in the 3D tortuosity values for all four lobes between the two populations. The positive z-score values indicated that the values were higher for control subjects than for Alzheimer’s patients (see Table 2).

**Table 1:** $\tau _{3D}$ values for each brain lobe

ID	Diagnosis	Frontal lobe	Occipital lobe	Parietal lobe	Temporal lobe
1	AD	67.4	31.8	59.7	36.6
2	AD	67.1	26.5	59.3	37.7
3	AD	67.7	30.1	54.5	38.0
4	Control	66.5	28.6	56.1	39.9
5	AD	63.8	35.0	55.9	36.6
6	Control	70.2	38.9	58.9	35.9
7	Control	68.5	33.3	59.0	43.1
8	AD	62.8	35.9	57.7	34.1
9	Control	75.3	34.8	60.7	40.2
10	AD	72.8	30.7	59.1	35.1
11	AD	68.6	30.2	54.8	36.4
12	AD	75.8	35.8	62.5	39.2
13	AD	67.2	30.1	57.0	38.7
14	AD	63.9	32.2	45.2	35.9
15	AD	67.0	32.0	57.1	36.7
16	AD	62.2	30.4	47.0	32.6
17	AD	69.1	34.6	64.2	40.5
18	AD	74.7	31.0	63.7	42.9
19	AD	58.2	32.5	55.5	38.4
20	AD	72.3	32.5	62.9	39.4
21	AD	70.4	35.2	62.0	39.9
22	AD	59.2	32.1	54.4	34.3
23	Control	73.3	32.2	63.1	39.9
24	Control	77.0	34.4	62.1	42.4
25	AD	77.3	33.7	63.4	40.8
26	Control	64.6	36.9	64.0	39.8
27	AD	63.4	35.4	60.4	36.4
28	Control	63.4	35.4	60.4	36.4
29	Control	63.4	35.4	60.4	36.4
30	AD	69.2	33.7	61.6	40.7
31	AD	53.8	39.8	61.0	30.9
32	Control	66.6	34.2	68.5	35.9
33	AD	69.9	33.6	63.0	38.2
34	Control	74.4	34.7	59.8	47.8
35	Control	69.2	34.7	65.5	43.7
36	Control	76.2	41.3	70.0	44.7
37	Control	72.4	36.8	64.3	47.4
38	AD	78.9	36.1	63.4	45.0
39	Control	70.9	38.5	67.7	41.2
40	AD	70.9	38.5	67.7	41.2
41	AD	52.6	29.8	45.8	34.9
42	Control	65.8	32.1	61.4	50.1
43	AD	68.4	34.5	55.5	46.9
44	AD	68.4	34.5	55.5	46.9
45	Control	86.7	35.1	73.2	49.8
46	AD	69.9	41.5	65.7	47.0
47	Control	70.6	32.3	62.4	47.8
48	AD	63.0	36.6	62.1	39.8
49	Control	67.0	37.5	68.8	45.2
50	AD	69.0	34.1	63.0	45.1
51	Control	70.7	33.6	62.5	45.9
52	Control	74.0	32.4	62.2	46.0
53	Control	69.2	36.7	64.1	42.6
54	AD	59.4	35.1	58.3	39.6
55	Control	74.6	33.3	58.7	41.9
56	AD	74.6	33.3	58.7	41.9
57	AD	61.2	31.3	55.1	40.0
58	AD	67.1	30.1	53.9	39.6
59	AD	57.3	27.4	45.5	35.9
60	AD	70.4	32.9	63.3	46.3

**Table 2:** Wilcoxon test results for brain lobes

Control-AD patients	Frontal lobe	Parietal lobe	Occipital lobe	Temporal lobe
*p-values*	0.0286	0.031	0.002	0.003
*z-values*	2.190	2.160	3.117	2.996

To address the potential confounding effects of age and sex on morphometric brain measures we performed additional linear regression analyses, incorporating these chacteristics as covariates for each lobe. The results showed a statistically significant association between the diagnosis (AD or control) and tortuosity values for three out of the four lobes: frontal, parietal, and temporal. However, the association was not statistically significant for the occipital lobe after adjusting for age and sex, see Table 3.

**Table 3:** Effect of diagnosis on tortuosity values by lobe after accounting for age and sex

Lobe	*p*-value for Diagnosis
Frontal	0.0171
Occipital	0.0648
Parietal	0.0013
Temporal	0.0056

**Fig. 7:**
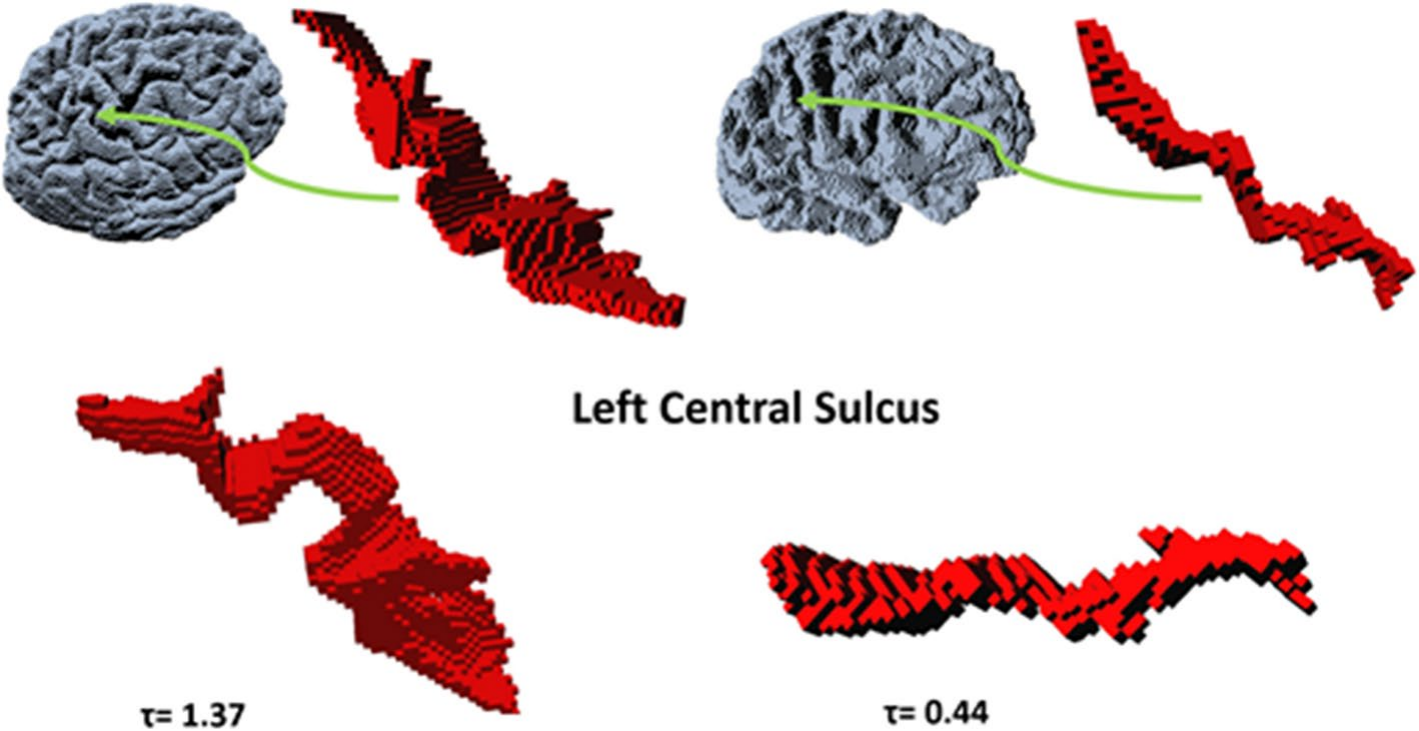
Two different central sulci of different subjects, both from the left hemisphere. Right, the voxelized volume of a patient with AD and the maximum value of tortuosity. Left, the voxelized volume of a control subject with the minimum value of tortuosity

We obtained the median (*M*) and standard deviation (std) $\tau _{3D}$ values for each population and each lobe. For the frontal lobe *M*= 68 with std=6 for AD patients, *M*= 71 with std=5 for control subjects. For the parietal lobe, *M*= 33 with std= 3 for AD patients, while *M*= 35 with std= 2. In the case of the occipital lobe *M*=59 with std= 5 for AD, and *M*= 62 with std= 4 for the control subjects. Finally, for the temporal lobe *M*=39 with std=4 for AD patients *M*= 43 with std=4 for control subject (see Table 1). After conducting the Wilcoxon test to analyze the differences in tortuosity values for the central sulcus between AD and control subjects, the results of this analysis are presented in Table 4. In this case the tortuosity values for the central sulcus of the left hemisphere resulted statistically significant $p=0.021$. When performing the linear regression analysis and accounting for age and sex again the tortuosity values for the central sulcus of the left hemisphere remain significant $p<0.05$. An example showcasing the 3D voxelized representation of the central sulcus is shown in Fig. 7.

**Table 4:** Wilcoxon test results for the central sulcus

AD patients-Control	Left hemisphere	Right-hemisphere
*p-values*	0.021	0.21
*z-values*	2.32	1.26

## Discussion

Although other methods are available for measuring the tortuosity of 3D objects, they are designed for specific phenomena and are not meant to describe the 3D morphological changes of volumetric objects. These methods focus on capturing changes in 3D trajectories, whereas our approach is specifically designed to measure the 3D morphological variations of volumetric objects. Consequently, our method cannot be compared with these other methods, establishing our proposal as the first one to measure the tortuosity of voxelized objects.

For example, Peyrega, Pardo-Alonso, and Gommes proposed a three-dimensional tortuosity measure to analyze porous media [41–43]. In [41], tortuosity is defined as the ratio between the geodesic distance from each voxel to two different subsets of the object and the Euclidean distance between them. This method calculates a tortuosity value for each voxel by connecting them through a geodesic path. However, it was specifically designed for 3D images of fibrous materials where entry and exit points are well-defined. Therefore, its applicability is limited to this type of volumetric object and cannot be extended to others. In [42] a comparison of four different methods for computing the geometrical tortuosity in infiltrated aluminum cellular materials is presented. The estimation methods compared were all based on the ratio between geodesic distance and Euclidean distance to define tortuosity. The difference among these methods was in the definition of the paths through the material. Gommes Cedric et al. [43] suggested measuring the tortuosity of porous materials using binary or grayscale reconstructions. They proposed two methods: the first approach involves directly comparing the geodesic and Euclidean distances calculated from any pore pixel to any limit of the tomogram. The second method is based on the geodesic reconstruction of the tomogram pore space, starting from any limits and taking into account its intensity variations.

The extension of the 2D SCC-based approach to 3D objects aimed to capture and quantify the variations in morphology that are indicative of the complexity of voxelized objects. The advantages of using the SCC-based approach for measuring and quantifying the morphological changes of volumetric objects are numerous. Firstly, the SCC is generated directly from the voxels, reducing sources of uncertainty and improving computation speed. Secondly, this approach can be applied to any voxelized object, making it widely applicable in various fields. Finally, the presented applications demonstrate possible methods for estimating $\tau _{3D}$, which can contribute to a more detailed description of different brain structures.

This work presents a novel to quantify the morphological changes in the brain’s folding structure attributable to Alzheimer’s disease (AD). Previous studies primarily focused on variations in sulcal width and cortical thinning as key descriptors of AD-related morphological changes. In contrast, our study introduces tortuosity as a descriptor that offers a different way to understand these variations. By estimating tortuosity across different brain lobes and the central sulcus, we demonstrate its potential in differentiating between AD patients and control subjects. Our findings reveal that tortuosity values (3D) are generally higher in control subjects, suggesting a decrease in the complexity of brain surface folding in AD patients.

These changes in tortuosity provide insights beyond traditional measures such as cortical thickness or volume loss. They reflect the non-isotropic nature of neurodegenerative changes in AD, where certain brain regions exhibit more pronounced morphological alterations. The decrease in tortuosity observed in AD patients aligns with the known phenomena of cortical thinning and widening of sulci. Our findings suggest that alterations in tortuosity capture the essence of how AD impacts brain morphology. This new understanding of tortuosity’s relationship with AD mechanisms underscores its potential value in early detection and monitoring of the disease’s progression.

## Conclusions

We introduced a new method to describe the shape of 3D voxelized objects, called $\tau _{3D}$. This method is based on the SCC approach originally proposed by Bribiesca for 2D curves [16]. $\tau _{3D}$ is computed as the normalized sum of all the slope chain elements for each filtered contour in every slice, and in the *X*, *Y*, and *Z* directions (Eq. 8).

To ensure that the computed tortuosity value corresponds more closely to the actual theoretical value, we filtered out the effect of the stair-stepping artifact on the 3D tortuosity measure. Also, we conducted a thorough methodological validation of the $\tau _{3D}$ with a series of controlled experiments. This validation establishes the accuracy and scale invariance of $\tau _{3D}$. However, validating $\tau _{3D}$ in a clinical scenario presents significant challenges. These challenges stem mainly from the practical difficulties associated with directly accessing brain structures.

The new $\tau _{3D}$ measure proposed in this study enables a quantitative characterization and comparison of the morphology of brain structures such as gray matter and central sulci. When analyzing the subjects of the MIRIAD database, the study found that the values of $\tau _{3D}$ obtained for the central sulci have the potential to serve as a biomarker for Alzheimer’s disease. Specifically, measuring the 3D tortuosity for the left central sulci showed statistically significant differences between patients and control subjects.

The potential applications of the proposed morphological feature are vast, particularly in medical imaging, where irregular shapes often hold important information. Our primary contribution is the extension of the two-dimensional tortuosity definition to three-dimensional space and the promising results it yields as a biomarker for neurodegenerative diseases. Future research should extend these findings to other databases containing neurodegenerative diseases and explore longitudinal studies.

## Data Availability

Data used to prepare this article were obtained from the public MIRIAD database (https://www.ucl.ac.uk/drc/research/methods/minimal-interval-resonance-imaging-alzheimers-disease-miriad).
